# Carbonic Anhydrase 3 Overexpression Modulates Signalling Pathways Associated with Cellular Stress Resilience and Proteostasis

**DOI:** 10.3390/ijms262412064

**Published:** 2025-12-15

**Authors:** Yezhou Yu, Merrina Anugraham, Tony Blick, Arutha Kulasinghe, Louise M. Sternicki, Giovanna Di Trapani, Sally-Ann Poulsen, Daniel Kolarich, Kathryn F. Tonissen

**Affiliations:** 1 School of Environment and Science, Griffith University, Brisbane, QLD 4111, Australia; 2 Institute for Biomedicine and Glycomics, Griffith University, Gold Coast, QLD 4215, Australia; 3 Frazer Institute, Faculty of Health, Medicine and Behavioural Sciences, The University of Queensland, Brisbane, QLD 4102, Australia; 4 Queensland Spatial Biology Centre, Wesley Research Institute, The Wesley Hospital, Brisbane, QLD 4066, Australia; 5 School of Environment and Science, Griffith University, Gold Coast, QLD 4215, Australia

**Keywords:** carbonic anhydrase 3, autophagy, hypoxia, RNA sequencing, proteomics, RANBP2, neurodegenerative disease, proteostasis, HSPA8, HSPA1A

## Abstract

Carbonic anhydrase 3 (CA3) exhibits low enzymatic activity compared to other CA isoforms but contains two surface-exposed cysteine residues that undergo glutathionylation under oxidative stress. Highly expressed in muscle tissue, CA3 has been implicated in cellular protection, particularly through interactions with Bcl2-Associated Athanogene 3 (BAG3), modulating autophagy, while CA3 overexpression decreased hypoxia-induced apoptosis in cardiomyocytes. In this study, we investigated the impact of CA3 overexpression on cellular pathways in HEK293T, MDA-MB-231, and SVCT cells using RNA sequencing and proteomics. Gene Set Enrichment Analysis (GSEA) in HEK293T cells revealed the down-regulation of pathways related to protein synthesis, RNA processing, Roundabout signalling, selenocysteine-metabolism, and suppression of neurodegenerative disease-associated pathways. Human breast epithelial cell lines under normoxia and hypoxia showed down-regulation of similar pathways, although notably, hypoxic conditions also suppressed interferon α/β signalling. Proteomic analysis in HEK293T cells using HaloTag pull-down experiments identified putative novel CA3 binding partners, including heat shock 70 kDa proteins 1 and 8, and ribosomal protein S2 (RPS2). RANBP2 protein was consistently up-regulated after CA3 overexpression, irrespective of the presence of CA3 surface-exposed cysteines and HaloTag orientation. These findings suggest that CA3 modulates key cellular processes beyond its enzymatic role, contributing to stress resilience through pathway-level regulation and protein interactions, potentially impacting autophagy and neurodegenerative disease.

## 1. Introduction

Carbonic anhydrases (CA) are zinc metalloenzymes that were first identified in the 1930s [1,2]. They catalyze the reversible hydration of carbon dioxide (CO_2_) to bicarbonate (HCO_3_^−^) and a proton (H^+^), which aids CO_2_ transportation and pH homeostasis. Currently, a total of 12 catalytically active CA isoforms have been identified, with CA2 characterized as the most active CA isoform (K_cat_ 1.4 × 10^6^ s^−1^) [3] and CA3 as the least active isoform (K_cat_ 1.3 × 10^4^ s^−1^) [4]. CA2 and CA3 share 62% amino acid sequence homology and have similar three-dimensional structures. The marked difference in their catalytic activity arises from a steric constraint in the active site of CA3, which restricts substrate access due to the presence of a phenylalanine residue at position 198, whereas CA2 has a leucine at the equivalent position [5]. CA3 has a 10-fold increase in its catalytic activity after replacing Phe198 with a leucine [5]. Lys64 is another contributor to the low activity of CA3, as the residue at the equivalent position in other isoforms is often a histidine that acts as a proton shuttle [6].

CA3 was first identified in 1976 as a muscle-specific CA isoform [7] and is closely associated with many muscular diseases [8,9,10,11,12]. Despite its low catalytic activity for the hydration of CO_2_, CA3 was reported to have antioxidant activities due to its two surface-exposed cysteine residues (Cys182 and Cys187), which can be glutathionylated under oxidative stress [13,14]. Among mammalian CAs, only CA7 shares these cysteines with CA3 [15]. CA3 overexpression can promote cell proliferation and make cells significantly more resistant to H_2_O_2_ treatment [16]. CA3 also serves in a protective role in rat nucleus pulposus and osteocyte cells [17,18].

Few functional studies on CA3 have been reported although, recently, two proteins that bind to CA3 have been identified. CA3 was found to be a binding partner of Bcl2-Associated Athanogene 3 (BAG3) in myasthenia gravis—a chronic autoimmune neuromuscular disorder characterized by weakness in skeletal muscles [9]. One pathogenic mechanism of myasthenia gravis involves the endocytosis of cholinergic receptor nicotinic (CHRN). Overexpression of CA3 inhibits autophagy through its interaction with BAG3, thereby attenuating CHRN degradation [9]. CA3 was also found to interact with squalene epoxidase (SQLE), a key enzyme implicated in non-alcoholic steatohepatitis—an advanced and inflammatory form of non-alcoholic fatty liver disease characterized by hepatocyte injury, inflammation, and fibrosis [19]. The SQLE-CA3 complex activates proinflammatory signalling pathways such as interleukin 6, tumour necrosis factor-α, and NF-κB pathways which have also been implicated in cancer development [19]. CA3 was also reported to promote epithelial–mesenchymal transition (EMT) [10], which is related to cancer invasiveness and metastasis [20]. Besides these, CA3 protects cells from hypoxia-induced apoptosis in cardiomyocytes [21] and has been proposed to activate the PI3K/AKT/mTOR pathway [20,21].

However, CA3’s cellular characteristics remain poorly defined and the binding mechanisms between CA3 and its protein binding partners are still unknown. In addition, how CA3 influences downstream pathways has only begun to be investigated. In this paper, we aimed to identify CA3-associated signalling pathways and unknown binding partners. Other studies have reported that CA3 overexpression resulted in changes to the cellular phenotype, but experimental approaches were focused on specific signalling pathways or targeted specific disease conditions [9,19,21]. In our current study, we utilized RNA sequencing to elucidate the effects of CA3 overexpression on cellular pathways in different cell types with the aim to further delineate CA3’s potential functions. Proteomics was used to identify new CA3 binding partners and to investigate how CA3 overexpression affects the expression pattern of other proteins.

## 2. Results

### 2.1. CA3 Overexpression and Transcriptomics Study

To better understand the effects of CA3 on cellular behaviour, RNA sequencing was conducted. Tag-free CA3 was overexpressed in HEK293T, MDA-MB-231, and SVCT cell lines. HEK293T was chosen due to its high transfection efficiency. Since CA3 has been reported to affect the EMT [10], the breast adenocarcinoma cell line MDA-MB-231, which is often used for cell migration and EMT studies [22], and SVCT, derived from non-cancerous breast epithelia [23], were used. For MDA-MB-231 and SVCT, CA3 overexpression was assessed under both normoxia (21% O_2_) and hypoxia (0.1% O_2_), due to the known link between hypoxia and EMT [24].

### 2.1.1. Differential Gene Expression

**Figure 1:**
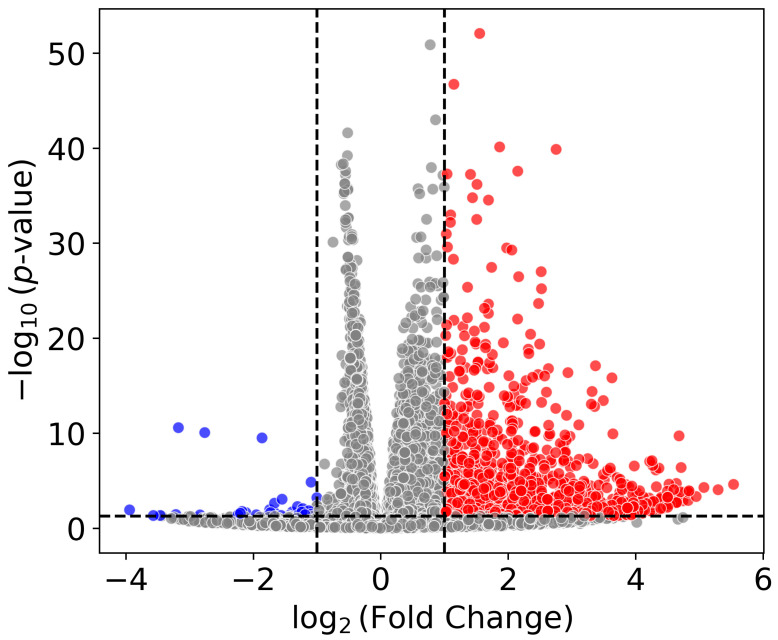
Volcano plot illustrating differentially expressed genes in HEK293T after CA3 overexpression under normoxia (N = 3). mRNA was extracted for sequencing 24 h after the transfection with pCA3-IRES2-EGFP or the pIRES2-EGFP control. After alignment and feature-counting, DESeq2 was used for paired tests. A *p*-value cutoff of 0.05 was used and is shown as a horizontal dashed line. An absolute log2-fold change threshold of 1 was used (vertical dashed lines). Due to homology with the plasmid sequence, CA3 and antisense-CA3 were excluded from the analysis. Up- and down-regulated genes are highlighted in red and blue, respectively.

RNA-Seq was performed on mRNA generated from each cell line under the stated conditions (N = 3). Differential Gene Expression (DGE) analysis was conducted, with transcript reads aligned to the reverse strand of National Center for Biotechnology Information (NCBI) reference genome GRCh38.p14. Subsequently, the alignment results were processed using featureCounts (version 2.0.1) [25] to obtain the reverse-strand feature counts. A paired DGE analysis was performed using DESeq2 (version 1.46.0) [26], providing log_2_-fold change, *p*-value, and adjusted *p*-value. In HEK293T cells under normoxia, CA3 overexpression resulted in the up-regulation of 1462 genes and the down-regulation of 39 genes (Figure 1).

**Figure 2:**
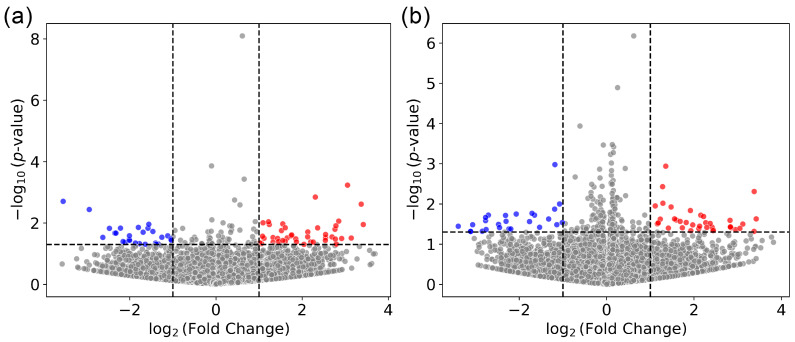
Volcano plot illustrating differentially expressed genes in breast cells (MDA-MB-231 and SVCT) after CA3 overexpression under (a) normoxia (21% oxygen) (N = 3) or (b) hypoxia (0.1% oxygen) (N = 3). mRNA was extracted for sequencing 24 h after the transfection with pCA3-IRES2-EGFP or the pIRES2-EGFP control. After alignment and feature-counting, DESeq2 was used for paired tests. A *p*-value cutoff of 0.05 was used and is shown as a horizontal dashed line. An absolute log2-fold change threshold of 1 was used (vertical dashed lines). Due to the homology with plasmid sequence, CA3 and antisense-CA3 were excluded from the analysis. Up- and down-regulated genes are highlighted in red and blue, respectively.

Data for the two breast epithelial cell lines (MDA-MB-231 and SVCT) were combined, and a paired analysis was performed using DESeq2. Under normoxia, 42 genes were found up-regulated after CA3 overexpression and 27 genes were found down-regulated (Figure 2a). Under hypoxia, 37 genes were found up-regulated and 26 were found down-regulated after CA3 overexpression (Figure 2b).

### 2.1.2. Pathway Analysis

Pathway analysis was employed to establish a comprehensive understanding of how CA3 influences cellular behaviour. More importantly, pathway analysis can often robustly detect pathway-level effects even when the individual genes within identified pathways lack statistical power. Gene Set Enrichment Analysis (GSEA) was used, utilizing ranked gene lists as input and providing normalized enrichment scores (NES), which offer a directional perspective on pathway modifications. A positive NES value signifies up-regulation, while a negative value indicates down-regulation. As NES do not imply statistical significance, a false discovery rate (FDR) q-value cutoff was applied. To identify consistent impacts of CA3 overexpression, rather than specific effects on individual cell lines, the results were combined from the two breast epithelial cell lines, MDA-MB-231 and SVCT.

Genes were ranked based on their directional log_10_ *p*-values, converted to a positive number for genes with a fold change >1. Subsequently, the ranked gene list was analyzed using GSEApy (version 1.1.9) [27] and the Reactome [28] and KEGG [29] databases.

**Figure 3:**
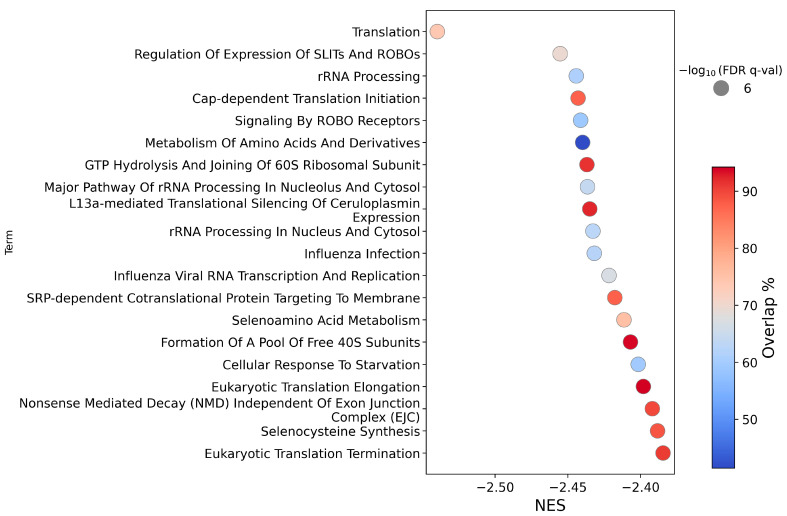
Circle plot illustrating the top 20 enriched pathways (*y*-axis), ranked by normalized enrichment score (NES, *x*-axis), from a Gene Set Enrichment Analysis (GSEA) of a ranked list of differentially expressed genes in HEK293T cells after CA3 overexpression under normoxia, using the Reactome database. Circle size reflects statistical significance (all pathways have −log10 FDR q-values at 6 due to the calculation limit), and colour indicates the pathway coverage (%). FDR q-val is a permutation-derived false discovery rate calculated using GSEApy.

Down-regulation of pathways was found by enrichment analysis of both the Reactome and KEGG databases for HEK293T CA3 overexpression. Most of the top 20 down-regulated Reactome pathways (Figure 3) relate to protein synthesis and RNA processing. Suppression was also observed for two pathways related to each of roundabout (ROBO) signalling and seleno-metabolism/synthesis. All top 20 pathways showed a strong statistical significance (−log_10_ FDR q-values at a maximum of the calculation limit of 6). Incomplete pathway coverage was due to zero read counts for some genes in the RNA sequencing results.

**Figure 4:**
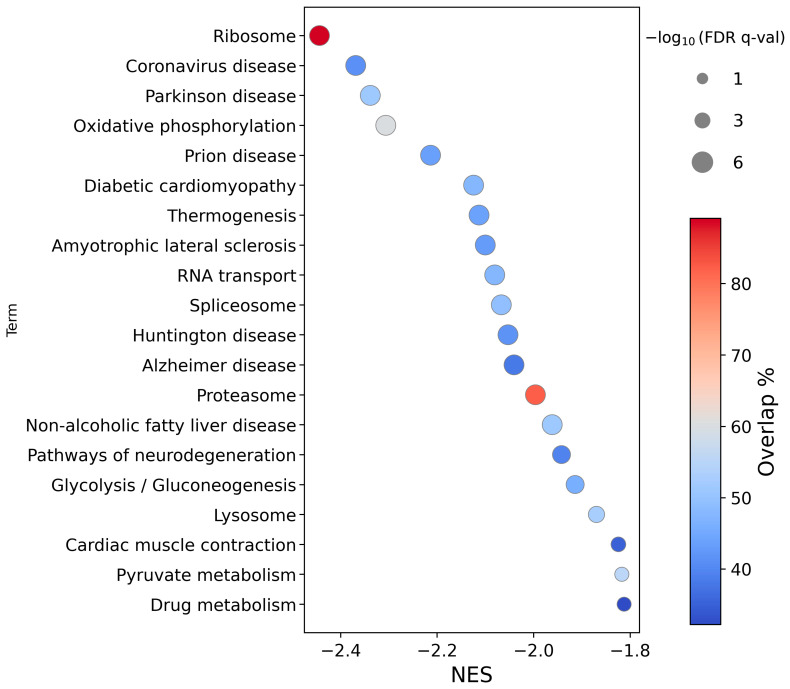
Circle plot illustrating the top 20 enriched pathways (*y*-axis), ranked by normalized enrichment score (NES, *x*-axis), from a Gene Set Enrichment Analysis (GSEA) of a ranked list of differentially expressed genes in HEK293T cells after CA3 overexpression under normoxia, using the KEGG database. Circle size reflects statistical significance (−log10 FDR q-values have an upper limit of 6 due to the calculation limit), and colour indicates the pathway coverage (%). FDR q-val is a permutation-derived false discovery rate calculated using GSEApy.

KEGG-based enrichment analysis (Figure 4) revealed negative enrichment of pathways primarily related to the neurodegenerative diseases; Parkinson’s, Huntington’s, Alzheimer’s, amyotrophic lateral sclerosis (ALS), and prion. Concordant with the Reactome-based results, “Ribosome” was the most down-regulated KEGG pathway. Other affected pathways included “Oxidative Phosphorylation”, “Non-alcoholic Fatty Liver Disease”, and “Diabetic Cardiomyopathy”. The top 14 pathways have −log_10_ FDR q-values at the maximum of the calculation limit, demonstrating strong statistical significance.

**Figure 5:**
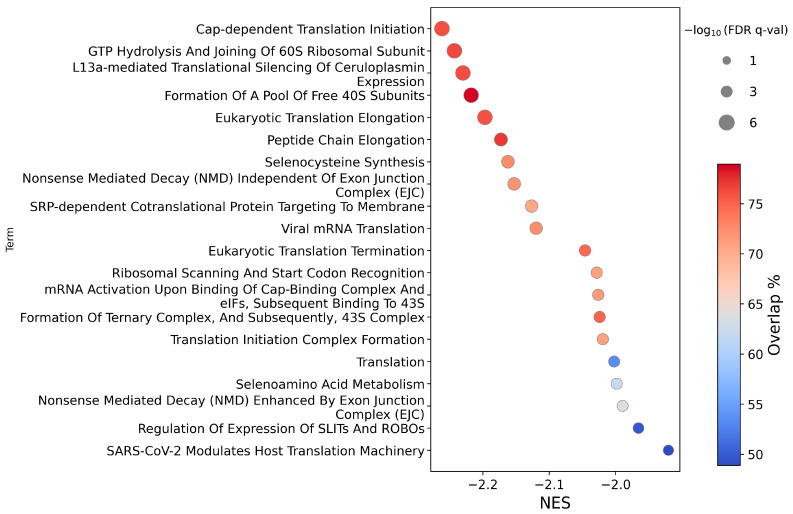
Circle plot illustrating the top 20 enriched pathways (*y*-axis), ranked by normalized enrichment score (NES, *x*-axis), from a Gene Set Enrichment Analysis (GSEA) of a ranked list of differentially expressed genes in MDA-MB-231 and SVCT cells after CA3 overexpression under normoxia, using the Reactome database. Circle size reflects statistical significance (−log10 FDR q-values have an upper limit of 6 due to the calculation limit), and colour indicates the pathway coverage (%). FDR q-val is a permutation-derived false discovery rate calculated using GSEApy.

CA3 overexpression under normoxia in breast epithelial cell lines (MDA-MB-231 and SVCT) revealed the down-regulation of Reactome pathways (Figure 5), but not for KEGG pathways. Concordant with the results for HEK293T cells, the most strongly suppressed pathways relate to protein synthesis. Pathways notably include the same two pathways involved in seleno-metabolism/synthesis that were found for HEK293T cells, along with one of the ROBO signalling pathways.

**Figure 6:**
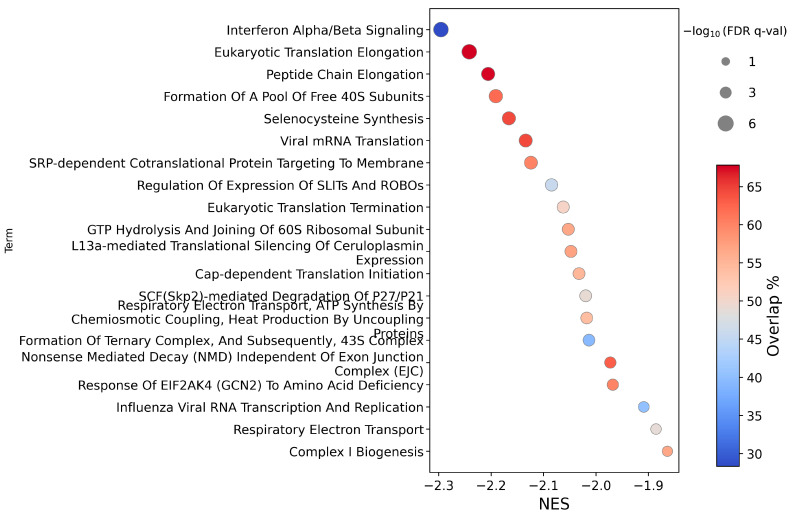
Circle plot illustrating the top 20 enriched pathways (*y*-axis), ranked by normalized enrichment score (NES, *x*-axis), from a Gene Set Enrichment Analysis (GSEA) of a ranked list of differentially expressed genes in MDA-MB-231 and SVCT cells after CA3 overexpression under hypoxia, using the Reactome database. Circle size reflects statistical significance (−log10 FDR q-values have an upper limit of 6 due to the calculation limit), and colour indicates the pathway coverage (%). FDR q-val is a permutation-derived false discovery rate calculated using GSEApy.

CA3 overexpression under hypoxia in breast cell lines (MDA-MB-231 and SVCT) largely mirrored the Reactome results under normoxia for protein synthesis, selenocysteine synthesis, and ROBO signalling pathways (Figure 6). Unique to breast cell lines under hypoxia, though, was the marked down-regulation of interferon α/β signalling. For the KEGG-based pathway analysis, “Ribosome” was found to be down-regulated under hypoxia.

### 2.2. CA3 HaloTag Experiments

To discover previously undescribed CA3 binding partners, four HaloTag-fused CA3 constructs were created, comprising a N- or C-terminus tag on wild type CA3 (CA3 WT) and a mutant CA3 with both surface-exposed cysteines mutated to serine (mutCA3) [30]. Tagged mutCA3 was included to study the role of CA3’s surface-exposed cysteine residues in protein–protein interactions. The HaloTag plasmid constructs were transfected into HEK293T cells, chosen due to their high transfection efficiency. HaloTag vector only was used as the control.

#### 2.2.1. Protein Pull-Down Assays

**Figure 7:**
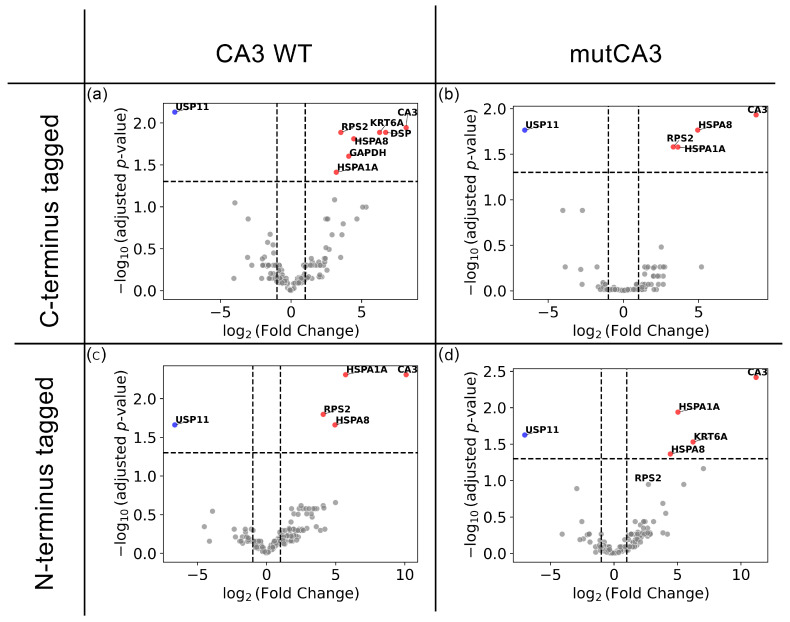
HEK293T cells were transfected with (**a**) C-terminus-tagged CA3 WT, (**b**) C-terminus-tagged mutCA3, (**c**) N-terminus-tagged CA3 WT and (**d**) N-terminus-tagged mutCA3. Proteins bound to CA3 were retrieved and analyzed by mass spectrometry (MS). MSFragger was used for paired tests between the control and CA3 overexpression groups and plotted as volcano plots with log2-fold change as the *x*-axis and −log10 adjusted *p*-value as the *y*-axis. An adjusted *p*-value cutoff of 0.05 was used and is shown as a horizontal dashed line. The adjusted *p*-value is calculated by Benjamini–Hochberg correction of *p*-values. An absolute log2-fold change threshold of 1 was used (vertical dashed lines). Proteins that are significantly up- and down-regulated due to CA3 or mutCA3 are highlighted in red and blue, respectively.

Heat shock 70 kDa proteins 1 and 8 (HSPA1A and HSPA8) were observed to be elevated in all CA3 pull-down samples (Figure 7), suggesting interactions that are independent of the surface-exposed cysteine residues. Ubiquitin specific protease 11 (USP11) was down-regulated in all CA3 transfected samples, implicating nonspecific binding to the HaloTag or affinity beads used for the pulldown. Ribosomal protein S2 (RPS2) was present in three of the CA3-pulldown samples, and detected as markedly changed in the fourth comparison, although not reaching statistical significance. Keratin 6A (KRT6A) was found with C-terminus-tagged CA3 WT and N-terminus-tagged mutCA3 and as a known contaminant in proteomics analysis [31]. Thus, it is unlikely that KRT6A is a specific CA3 interaction partner.

#### 2.2.2. Proteomic Alterations

**Figure 8:**
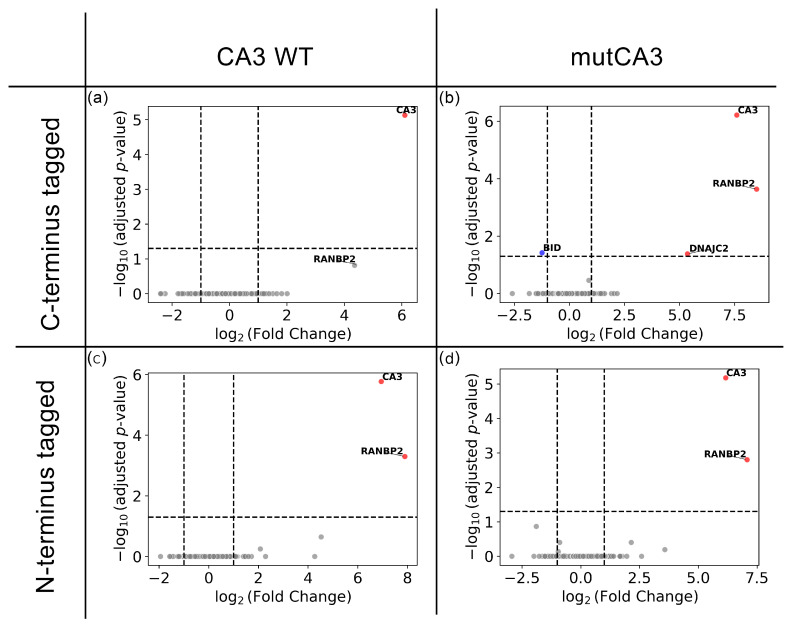
HEK293T cells were transfected with (**a**) C-terminus-tagged CA3 WT, (**b**) C-terminus-tagged mutCA3, (**c**) N-terminus-tagged CA3 WT and (**d**) N-terminus-tagged mutCA3. Cell lysates were analyzed by mass spectrometry (MS). MSFragger was used for paired tests between the control and CA3 overexpression groups and plotted as volcano plots with log2-fold change as the *x*-axis and −log10 adjusted *p*-value as the *y*-axis. An adjusted *p*-value cutoff of 0.05 was used and is shown as a horizontal dashed line. The adjusted *p*-value is calculated by Benjamini–Hochberg correction of *p*-values. An absolute log2-fold change threshold of 1 was used (vertical dashed lines). A total of 1185 proteins were identified across all samples. Proteins that are significantly up-regulated due to CA3 or mutCA3 overexpression are highlighted in red.

In addition to the CA3 pull-down proteins, the whole lysates after CA3 overexpression were also digested and analyzed by LC-MS, with 1185 proteins identified in total across all samples (Supplementary Table S1). RAS-related nuclear protein binding protein 2 (RANBP2) was found significantly up-regulated in three comparisons and detected as markedly changed in the fourth comparison, although not reaching statistical significance. This result is indicative that binding is not affected by the presence of surface-exposed cysteines or HaloTag orientation (Figure 8). Since RANBP2 was not identified amongst the CA3 pull-down proteins and the RNA sequencing analysis revealed that the mRNA level of RANBP2 was unaffected by CA3 overexpression (−0.03 log_2_-fold change with a *p*-value of 0.66), the >20-fold increase in its protein level across all four experimental groups likely indicates a change in RANBP2’s steady state level. Of the 1185 proteins identified, only CA3 and alpha-cardiac actin (ACTC1) had differentially expressed RNA.

## 3. Discussion

In this study, we utilized RNA sequencing and proteomics methods to systematically assess how CA3 overexpression affected cell signalling pathways, the impact on protein levels, and to identify potential unknown CA3 binding partners.

CA3 overexpression resulted in a marked increase in the steady state level of RANBP2, a component of the nuclear pore complex [32], and may be the mechanism responsible for the observed down-regulation of pathways related to the following neurodegenerative diseases: Parkinson’s, Huntington’s, Alzheimer’s, ALS and prion diseases. RANBP2 plays multiple roles in nuclear-cytoplasmic transport, SUMOylation, and protein homeostasis [33]. RANBP2 both facilitates and provides target specificity for SUMO-conjugation, a post-translational modification that affects the stability, localization, and activity of proteins, including those implicated in Parkinson’s (α-synuclein), Alzheimer’s (amyloid precursor protein (APP), tau), and Huntington’s (huntingtin) diseases [34,35,36]. Mutation or dysregulation of *RANBP2* can lead to the development of a range of human pathologies, including these neurodegenerative diseases, as well as cancer [37]. For example, RANBP2 protein levels are lower in hippocampal extracts from ME7 mice, a prion model for human neurodegenerative disease [38]; additionally, RANBP2 down-regulation in motor neurons disrupts multiple essential cellular pathways and directly induces ALS-like neurodegenerative pathology in vivo [39]. The ubiquitin-protein ligase encoded by the parkin (*PRKN*) gene can cause autosomal recessive Parkinson’s disease and has been shown to specifically bind to and target RANBP2 for proteasomal degradation [40]. Therefore, CA3-induced up-regulation of RANBP2 may exert a neuroprotective effect by enhancing SUMOylation of disease-related proteins, stabilizing nuclear transport, and modulating protein degradation mechanisms. Up-regulation of RANBP2 can also accelerate cell cycle progression and promote proliferation [41,42,43], which matches the reported effects of CA3 overexpression [10,21]. While the list of indirect mechanisms that can lead to elevated protein steady state levels is extensive, we speculate that the observed increase in RANBP2 protein may be due to either improved chaperone efficiency and/or decreased RANBP2 degradation, given that two heat shock proteins were found to be CA3 binding partners and neurodegenerative disease pathways were found to be down-regulated, which include processes that specifically degrade RANBP2.

The observed down-regulation of protein synthesis and RNA processing pathways as a consequence of CA3 overexpression, along with RPS2 being identified as a binding partner, may also offer neuroprotection through several synergistic mechanisms, including the reduction in toxic protein accumulation, suppressing pathways that lead to neurodegenerative disease and decreasing cellular stress [44,45,46]. This proposed function aligns with the reported role of CA3 as being protective for cells under stress conditions [16,17,18]. While attenuating cellular proteostasis in response to stress is believed to be beneficial in terms of providing neuroprotection, long term impairment could interfere with neuron survival [47].

**Figure 9:**
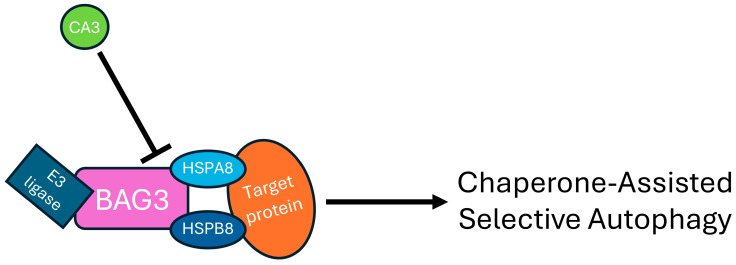
Proposed role of CA3 in chaperone-assisted selective autophagy (CASA). The chaperones HSPB8 and HSPA8 recognize misfolded or damaged proteins, while the co-chaperone BAG3 mediates formation of the CASA complex by also recruiting E3 ligases to initiate autophagy. CA3 interacts with both BAG3 and HSPA8 within the CASA complex to modulate its activity, with the BAG3–CA3 interaction previously shown to exert an inhibitory effect on autophagy.

CA3 has previously been implicated in autophagy-mediated protein degradation, offering protection against the neuromuscular disorder, myasthenia gravis, through its interaction with BAG3 [9]. The BAG3-CA3 complex prevents the endocytosis and degradation of CHRN, thereby preserving its function. While our CA3 pull-down assays did not detect BAG3, we did identify heat shock 70 kDa protein 8 (HSPA8), a known binding partner of BAG3 [48,49], as a putative CA3-interacting protein. The BAG3–HSPA8 complex orchestrates chaperone-assisted selective autophagy (CASA), a pivotal pathway for maintaining protein homeostasis in smooth and skeletal muscles [48,50]. Defects in BAG3 function have been associated with various muscle and cardiac pathologies, including muscular dystrophy [51,52,53]. In muscle cells, CASA targets damaged Z-disc proteins via a mechanism where HSPA8 recognizes misfolded substrates, and BAG3 mediates formation of the CASA complex, comprising HSPA8, HSPB8, and E3 ubiquitin ligases to initiate selective degradation [48,50,54]. CA3 itself is a muscle-enriched protein with elevated expression levels in response to physical exercise [55] and muscle injury [11]. Given this context, CA3 may function as a structural or regulatory component of the CASA machinery (Figure 9), facilitating targeted removal of damaged protein complexes in the muscle, while protecting other proteins. This hypothesis extends logically to neuronal systems, since all of the neurodegenerative disease-associated pathways found down-regulated in this study are related to protein aggregation. CASA-like mechanisms appear to be activated to clear these toxic aggregates [56,57,58,59,60], including BAG3, which has previously been linked to autophagy in an ALS model [49]. However, CA3’s role in neurons remains to be elucidated.

We also identified the heat shock protein HSPA1A [61] as a putative CA3 binding partner. Reduction in the levels of either HSPA1A or HSPA8 has been shown to lead to an increase in the levels of the neurodegenerative proteins: tau, superoxide dismutase 1, and a-synuclein [62,63]. Alterations of HSPA8 activity have been linked to both neurodegenerative and neuropsychiatric diseases, such as neuroaxonal dystrophy, schizophrenia, Parkinson’s and ALS [64,65,66,67], with both HSPA1A and HSPA8 associated with neuroprotection.

CA3 overexpression also resulted in down-regulation of the roundabout guidance receptor 1 (ROBO1) signalling pathway, which under normal conditions is activated by the binding of SLIT ligands and plays several important intracellular functions. ROBO1 signalling guides axon navigation and neuronal cell migration during development, modulates cytoskeletal dynamics to regulate cell adhesion and motility, and inhibits mTOR pathway activity, resulting in enhanced autophagy [68,69]. Impairment of ROBO signalling in cancer cells was shown to induce hyperactivation of mTORC1 and suppresses autophagy [69], which is consistent with reports that CA3 overexpression activates the mTOR pathway [21] and inhibits autophagy [9,19].

SLIT/ROBO signalling has also been linked to neurological diseases. Dysregulation of SLIT/ROBO signalling has been proposed as a disease mechanism for both idiopathic and monogenic Parkinson’s [70]. APP, the aggregation of which causes Alzheimer’s disease, is also a receptor for SLIT, mediating neural circuit formation via axon guidance [71]. Low SLIT/ROBO signalling associates with ALS progression, whereas elevated expression can have a protective effect [71]. Genetic evidence links ROBO receptors to the following neurodevelopment disorders: autism, schizophrenia, dyslexia, horizontal gaze palsy with progressive scoliosis syndrome, intellectual disability, strabismus, and psychopathy [72,73,74,75,76,77,78].

CA3 has been previously linked to protecting cells from oxidative stress [13,14,16,17,18,21]. In this study, selenocysteine-related pathways were down-regulated after CA3 overexpression. These pathways are involved in the synthesis of glutathione peroxidase (GPX) and thioredoxin reductase (TrxR). Down-regulation in these two pathways indicates a suppression of GPX and TrxR synthesis, respectively. However, the mRNA and protein levels of the GPX and TrxR families of proteins largely remained unchanged. The down-regulation of the selenocysteine-related pathways may eventually impact the redox protein function [79], generating truncated protein variants that will be targeted for degradation.

‘Interferon a/b signalling’, which is known to be down-regulated by hypoxia [80], was found to be further down-regulated in response to CA3 overexpression under hypoxic conditions. While there is no reported connection between CA3 and interferon signalling, modulation of SLIT/ROBO signalling has been shown to impact macrophage polarization and interferon signalling [81].

Future research should focus on validating the newly identified CA3 binding partners and elucidating their cellular functions, particularly in relation to autophagy and neuroprotection. Additionally, the connection between CA3 and the signalling pathways revealed by RNA sequencing warrants further investigation, especially in the context of neurodegenerative disorders. Special attention should be given to how CA3 may influence the steady state protein levels of RANBP2 and, consequently, its diverse roles in cellular regulation.

## 4. Materials and Methods

### 4.1. Plasmids Used for Transfection

CA3 mRNA sequence [30] was cloned into pIRES2-EGFP (Clontech, Mountain View, USA) to express tag-free wild type CA3 (pCA3-IRES2-EGFP). Wild type CA3 and CA3, with both surface-exposed cysteine residues (Cys182 and Cys187) mutated (mutCA3), were cloned into the HaloTag vectors pFC14K and pFN21A (Promega, Madison, WI, USA), where the HaloTag is located on the C-terminal in pFC14K and N-terminal in pFN21A (pFC14K-CA3WT, pFC14K-mutCA3, pFN21A-CA3WT and pFN21A-mutCA3).

### 4.2. Cell Culture and Protein Overexpression

HEK293T (ATCC^®^ CRL-3216^TM^), MDA-MB-231 (ATCC^®^ HTB-26^TM^) [82], and SVCT [23] cell lines were kind gifts from Dr. Xiaoyi Chen (Griffith University, Brisbane, Australia), Professor E. Thompson (Queensland University of Technology, Brisbane, Australia), and Professor Kum Kum Khanna (Mater Research Institute, Brisbane, Australia), respectively.

Cells were grown at 37 °C with 5% CO_2_ under atmospheric oxygen conditions (21% O_2_) for normoxic incubation. For hypoxic conditions, cells were cultured at 37 °C with 5% CO_2_ and 0.1% O_2_ in a C-Chamber hypoxic growth chamber attached to the ProOx C21 model controller (Biospherix, New York, NY, USA). Dulbecco’s Modified Eagle Medium (DMEM) (Thermo Fisher Scientific, Waltham, MA, USA) with 10% fetal bovine serum (FBS) (Bovogen, Melbourne, Australia) was used as the complete growth media.

Lipofectamine 3000 Reagent (Thermo Fisher Scientific) was used for transfection. For RNA sequencing, approximately 0.3–0.4 million cells were seeded into 2 mL of complete growth media in each well of a 6-well plate. Cells were cultured at 37 °C overnight to let them attach. Two solutions were prepared on the second day. Solution 1 contained 5 µg plasmid DNA, 5 µL P3000 Reagent and 125 µL Opti-MEM medium. Solution 2 contained 5 µL Lipofectamine 3000 Reagent and 125 µL Opti-MEM. The two solutions were mixed and incubated at room temperature for 10 min and slowly added to the cells in triplicate. The plate was gently swirled and incubated at 37 °C before further experimentation. The transfection was scaled up to a 12 cm Petri dish for the proteomics study.

### 4.3. RNA Sequencing Sample Preparation and Data Normalization

RNA was extracted using the Monarch Total RNA Extraction and Isolation Kit (New England Biolabs, Ipswich, MA, USA). RNA samples were sent to the Griffith University DNA Sequencing Facility (GUDSF) (Nathan, QLD, Australia) for sequencing. Raw RNA sequencing data obtained from GUDSF were processed using STAR (version 2.7.10b) [83]; transcript reads were aligned to the reverse strand of the National Center for Biotechnology Information (NCBI) reference genome GRCh38.p14 (accessed on 3 May 2025). Subsequently, the alignment results were processed using featureCounts (version 2.0.1) [25] to obtain the reverse-strand feature counts.

### 4.4. Differential Gene Expression Analysis

Raw counts obtained from featureCounts [25] were normalized using DESeq2 (ver. 1.46.0) [26]. Samples were grouped accordingly, and paired statistical tests were performed to compare the different groups. Normalized counts, log_2_-fold changes, *p*-values, and adjusted *p*-values (FDR) were subsequently exported for downstream analysis. Volcano plots for data visualization were generated using Python’s (version 3.13.5) Matplotlib (version 3.10.0) and Seaborn libraries (version 0.13.2).

### 4.5. RNA Sequencing Pathway Analysis

All genes with valid fold change values in the DESeq2 results were ranked based on their directional log_10_ *p*-values. The ranked list was then imported into GSEApy (version 1.1.9) [27] and enriched against the Kyoto Encyclopedia of Genes and Genomes (KEGG) and Reactome databases, with permutation set to 10,000. The results were exported as a dot maps using Matplotlib and Seaborn libraries (Python).

### 4.6. Cell Transfection and HaloTag Pull-Down Protein Procedure

HEK293T cells were transfected in triplicate with the HaloTag plasmids. After overnight incubation, the media was discarded, and cells were harvested in ice-cold phosphate-buffered saline (PBS). Samples were centrifuged at 2000× *g* for 10 min and the supernatant was discarded. The cell pellets were stored at −80 °C for 30 min. The pellet was then thawed and lysed in 300 µL of Mammalian Lysis Buffer (Promega), followed by the addition of 6 µL of 50× Protease Inhibitor Cocktail. Samples were incubated on ice for 5 min and then the lysate was cleared by centrifugation at 14,000× *g* for 5 min. The supernatant (~300 µL) was transferred into a new tube and diluted with 700 µL of 1× Tris-buffered saline pH 7.5 (Promega). HaloLink Resin (200 µL, Promega) was spun down (800× *g*, 1 min) and washed with 800 µL of Resin Equilibration/Wash Buffer (Promega) for a total of 3 washes. The resin was centrifuged at 800× *g* for 2 min and the supernatant was discarded. The cleared and diluted cell lysate was added to the resin and incubated on a tube rotator at room temperature for 15 min. The sample was then centrifuged for 2 min at 800× *g* and the supernatant was discarded. The resin was washed four times with 1 mL of Resin Equilibration/Wash Buffer and then centrifuged at 800× *g* for 2 min. Then 50 µL of SDS Elution Buffer (Promega) was added to the resin and the sample was incubated at room temperature with shaking for 30 min. The sample was centrifuged for 2 min at 800× *g* and the supernatant was transferred into a new tube for downstream proteomics analysis.

### 4.7. Proteomics Sample Preparation and Protein Identification

Protein samples were processed using S-Trap micro spin columns (ProtiFi, Fairport, USA) and protein quantitated with a NanoDrop (Thermo Fisher Scientific). A total of 100 µg protein (11.5 µL protein sample) was diluted with 2× SDS lysis buffer (ProtiFi, Fairport, NY, USA) to a final volume of 23 µL. Samples were reduced by adding 1 µL of 120 mM DTT (Merck, Rahway, NJ, USA) and incubating at 55 °C for 15 min, followed by alkylation with 1 µL of 500 mM iodoacetamide (Merck, Rahway, NJ, USA) at room temperature for 10 min in the dark. The reaction was quenched and acidified with 2.5 µL of 27.5% phosphoric acid according to the manufacturer’s protocol before 165 µL of binding/wash buffer (ProtiFi) was added, and the mixture was loaded onto the S-Trap micro column and centrifuged at 4000× *g* for 30 s. The columns were washed by adding 150 µL of binding/wash buffer (ProtiFi) and centrifuging at 4000× *g* for 30 s. The flow-through was discarded, and the wash step was repeated twice. Columns were then centrifuged at 4000× *g* for 1 min to completely remove residual wash buffer. For digestion, 20 µL of digestion buffer (ProtiFi) containing trypsin was added to the columns, which were incubated overnight at 37 °C. Peptides were eluted in three sequential steps using 40 µL of elution buffer 1 (ProtiFi), 40 µL of elution buffer 2 (ProtiFi), and 40 µL of elution buffer 3 (ProtiFi). The eluates were combined and dried using a speedvac concentrator (Thermo Fisher Scientific). The dried peptide pellets were resuspended in 50 µL of 0.1% trifluoroacetic acid (TFA). The peptides were analyzed using an Orbitrap Fusion Tribrid mass spectrometer (Thermo Fisher Scientific), coupled to an UltiMate 3000 UHPLC fitted with a trap column (PepMap Neo C18, 5 mm × 300 µm, 5 µm particle size) and analytical reversed-phase C18 PepMap Neo (75 μm × 500 mm, 2 µm particle size) UHPLC column (Thermo Fisher Scientific) and PicoTip nanospray interface (New Objective, Littleton, MA, USA). The samples were injected onto the trap column in 0.1% TFA (Loading Solvent A) at a flow rate of 15 µL/min. The analytical column was first equilibrated in 99% solvent A (0.1% FA) at a flow rate of 300 nL/min and the peptides were separated over a linear gradient as follows: 1–3% solvent B (80% acetonitrile, 0.1% formic acid (FA)) from 0 to 8 min, 3–10% from 8 to 13 min, 10–30% from 13 to 63 min, 30–50% from 63 to 70 min, 50–95% from 70 to 72 min, and 95% from 72 to 73 min, before the column was re-equilibrated in 1% solvent B from 75 to 90 min.

### 4.8. Label-Free Quantitative (LFQ) Proteomics Analyses and Data Visualization

Proteins were identified using Proteome Discoverer (version 3.1.1.93, Thermo Scientific) and quantified using FragPipe-Analyst (version 1.22) [84]. For protein and peptide identification, the raw LC-MS/MS data were searched against the UniProtKB *Homo sapiens* proteome database (Proteome ID: UP000005640, downloaded 12 April 2016, 20 405 entries). The trypsin enzymatic cleavage at the C-terminus of amino acid residues lysine (K) and arginine (R) was selected with a maximum of two missed cleavages allowed, with peptide lengths ranging from 6 to 144 amino acids. Peptide precursor mass and fragment mass tolerances were set to 10 ppm and 0.02 Da, respectively. The search criteria included carbamidomethylation of cysteine residues (+57.0215 Da) as a static modification and variable modifications consisted of methionine oxidation (+15.9949 Da), acetylation (+42.011 Da) at N-terminus of protein, and deamidation (+0.984 Da) of asparagine. Data were filtered with appropriate false discovery rates for both the peptide and protein levels (FDR < 0.01), and peptide spectral matches (PSMs, delta Cn: 0.05) to ensure high confidence identifications.

For label-free quantitation, the data were searched against the human database protein sequences (reviewed sequences with isoforms; downloaded on 20 November 2025) using the MSFragger node (version 4.3) of FragPipe (version 23.1), with full tryptic cleavage specificity allowed for up to two missed cleavages and above-mentioned static and variable modifications. The IonQuant feature detector node (version 1.11.11) of FragPipe was utilized for high PSM confidence, with a minimum of two isotopes required for protein-level quantification. FDR filtering was applied by default (1% FDR) for all PSMs, peptides, and protein levels. For statistical analyses and differential expression analyses, FragPipe-Analyst was utilized, using peptide-wise linear models combined with the empirical Bayes method (limma package from R Bioconductor) to generate a list of differentially expressed peptides for each pairwise comparison. An adjusted *p*-value cutoff of 0.05, together with a log2-fold change of 1, was applied to determine differentially expressed peptides in each pairwise comparison. All data were normalized (variance stabilizing node) and imputed with Perseus-type imputation and Benjamini–Hochberg FDR correction for multiple comparison analyses.

## 5. Conclusions

In summary, CA3 overexpression appears to exert multifaceted protective effects across cellular systems, particularly in the context of neurological and metabolic diseases. The marked increase in the steady state level of RANBP2 suggests a potential mechanism by which CA3 may enhance SUMOylation and nuclear transport, thereby stabilizing proteins implicated in neurodegenerative disorders such as Parkinson’s, Alzheimer’s, and Huntington’s diseases. Concurrently, CA3’s influence on autophagy-related pathways, through interactions with HSPA8 and possibly the CASA complex, points to a role in maintaining protein homeostasis, especially in muscle and potentially neuronal tissues. Although CA3 overexpression led to the down-regulation of selenocysteine-related and RNA processing pathways, these changes may reflect adaptive responses that mitigate cellular stress and toxic protein accumulation. Collectively, these new findings support a model in which CA3 functions as a stress-responsive regulator, integrating protein quality control, autophagy, and neuroprotective mechanisms.

## Data Availability

The RNA sequencing datasets described in this study are openly available in the NCBI Gene Expression Omnibus (https://www.ncbi.nlm.nih.gov/geo/ (accessed on 26 November 2025)) with accession number GSE311117. The LC-MS proteomics data has been deposited to the ProteomeXchange Consortium via the PRIDE [85] partner repository (https://www.ebi.ac.uk/pride/ (accessed on 10 December 2025)) with the dataset identifiers PXD071664 and 10.6019/PXD071664, PXD071770 and 10.6019/PXD071770. Supplementary Table S2 lists the uploaded proteomics data files, together with their relevant dataset identifier.

## Supplementary Materials

The following supporting information can be downloaded at: https://www.mdpi.com/article/10.3390/ijms262412064/s1.

## Abbreviations

The following abbreviations are used in this manuscript:

ALS	Amyotrophic lateral sclerosis
APP	Amyloid precursor protein
BAG3	Bcl2-Associated Anthanogene 3
CA	Carbonic Anhydrase
CASA	Chaperone-assisted selective autophagy
CHRN	Cholinergic Receptor Nicotinic
CO_2_	carbon dioxide
DMEM	Dulbecco’s Modified Eagle Medium
DGE	Differential Gene Expression
EMT	Epithelial–mesenchymal transition
FA	Formic acid
FDR	False Discovery Rate
GPX	Glutathione Peroxidase
GSEA	Gene Set Enrichment Analysis
HSPA1A	Heat shock 70 kDa protein 1A
HSPA8	Heat shock 70 kDa protein 8
KEGG	Kyoto Encyclopedia of Genes and Genomes
KRT6A	Keratin 6A
MS	Mass spectrometry
mTOR	mechanistic Target Of Rapamycin
NCBI	National Center for Biotechnology Information
NES	Normalized Enrichment Score
NF-kB	Nuclear factor kappa-light-chain-enhancer of activated B cells
NPC	Nuclear Pore Complex
PBS	Phosphate-buffered saline
PRKN	Parkin
RANBP2	RAS-related nuclear protein binding protein 2
ROBO1	Roundabout guidance receptor 1
RPS2	Ribosomal protein S2
SQLE	Squalene Epoxidase
SUMO	Small Ubiquitin-like Modifier
TFA	Trifluoroacetic acid
TrxR	Thioredoxin Reductase
USP11	Ubiquitin Specific Protease 11
